# Results of a randomized controlled trial analyzing telemedically supported case management in the first year after living donor kidney transplantation - a budget impact analysis from the healthcare perspective

**DOI:** 10.1186/s13561-016-0141-3

**Published:** 2017-01-13

**Authors:** Klaus Kaier, Silvia Hils, Stefan Fetzer, Philip Hehn, Anja Schmid, Dieter Hauschke, Lioudmila Bogatyreva, Bernd Jänigen, Przemyslaw Pisarski

**Affiliations:** 1 Institute for Medical Biometry and Statistics, Faculty of Medicine and Medical Center – University of Freiburg, Stefan-Meier-Str 26, 79104 Freiburg, Germany; 2 Transplantation Center, Department of General and Visceral Surgery, Medical Center, Faculty of Medicine, University of Freiburg, Hugstetter Str 55, 79106 Freiburg, Germany; 3 Faculty of Economics, Aalen University, Beethovenstraße 1, 73430 Aalen, Germany

**Keywords:** Telemedicine, Cost-of-illness, Cost-benefit

## Abstract

**ᅟ:**

We analyze one-year costs and savings of a telemedically supported case management program after kidney transplantation from the perspective of the German Healthcare System. Recipients of living donor kidney transplantation (*N* = 46) were randomly allocated to either (1) standard aftercare or (2) standard aftercare plus additional telemedically supported case management. A range of cost figures of each patient’s medical service utilization were calculated at month 3, 6 and 12 and analyzed using two-part regression models.

In comparison to standard aftercare, patients receiving telemedically supported case management are associated with substantial lower costs related to unscheduled hospitalizations (mean difference: €3,417.46 per patient for the entire one-year period, *p* = 0.003). Taking all cost figures into account, patients receiving standard aftercare are associated, on average, with one-year medical service utilization costs of €10,449.28, while patients receiving telemedically supported case management are associated with €5,504.21 of costs (mean difference: € 4,945.07 per patient, *p* < 0.001). With estimated expenditures of €3,001.5 for telemedically supported case management of a single patient, we determined a mean difference of €1,943.57, but this result is not statistically significant (*p* = 0.128). Sensitivity analyses show that the program becomes cost-neutral at around ten participating patients, and was beneficial starting at 15 patients. Routine implementation of telemedically supported case management in German medium and high-volume transplant centers would result in annual cost savings of €791,033 for the German healthcare system.

Patients with telemedically supported case management showed a lower utilization of medical services as well as better medical outcomes. Therefore, such programs should be implemented in medium and high-volume transplant centers.

**Trial registration:**

DRKS00007634 (http://www.drks.de/DRKS00007634).

## Background

Successful solid organ transplantation can offer substantial improvements to patient quality of life. However, the recovery process after organ transplantations requires continuous effort and therapy to ensure and maintain satisfactory patient outcome and prevent graft loss [1]. Patient adherence to long-term medication is often insufficient for a variety of reasons, and many potential behavioral pitfalls have to be avoided [2–5].

Prevention of graft loss, beside its significance to individual patients, also represents a concern to the healthcare system as a whole due to the high number of patients on the German kidney waiting list and the permanent shortage of donor organs. In the literature there are different propositions on how to reduce occurrence of graft loss, however not all of them are equally effective in producing the desired results [6]. The continuing progress of communication technology provides healthcare professionals an expanding set of tools to potentially improve patient outcomes.

Our random controlled trial combines telemedically supported case management and monitoring of patient’s vital data with video teleconferences between the patient and the Transplantation Center Freiburg [7]. The trial was registered with the German Clinical Trials Register (www.DRKS.de) under the identification number DKRS00007634.

The results of our random controlled trial show significant improvements in medical and disease-specific outcomes over the first year after transplantation as described in detail in Schmid et al. [8].

But beyond this medical dimension, telemedically supported case management also has an economic perspective. On the one hand costs of medical treatment increased due to necessary investments into the new telemedicine infrastructure and hiring and training of specialized personnel. On the other hand cost savings can be achieved due to prevention of medical emergencies, elimination of unneeded doctor visits and specialist consultations, and more appropriate therapy decisions. With regard to the resulting net cost effects of telemedically supported intervention prior evidence is mixed and sometimes flawed [9–12]. The benefits of telemedicine in delivering effective care coordination have been confirmed in chronic disease conditions such as cardiac insufficiency [13–15]. A new broad German study focus on telemonitoring for COPD using routine data from a major German sickness fund clearly demonstrates positive effects on healthcare costs [16].

We advance the debate on routine implementation of telemedicine in the health system using a case study from the clearly delimited field of kidney transplantation’s aftercare. To best of our knowledge this is the first evaluation of a telemedicine and case-management intervention for transplantation patients with focus on economic (budget) impact in the German healthcare system, observing costs in the first year after transplantation. Furthermore we demonstrate that even if it is clear that a telemedicine intervention is dominant, i.e. preferable both medically and economically to standard care, the current reimbursement practice of German sickness funds still makes it difficult to implement the technique in a routine manner.

## Methods

### Data collection

A prospective, controlled, randomized and open project-study was realized as follows [7]: In September 2011, 50 patients who were scheduled for a living-donor kidney transplantation at the Transplantation Center Freiburg between October 2011 and March 2012 were randomly allocated to two groups receiving different aftercare programs during the first postoperative year.

The first group was offered standard aftercare in combination with telemedically supported case management. Daily, the patients completed a pre-defined medical questionnaire about their physical condition presented to them in their homes via an interactive web-based telemonitor. These data were monitored by medical staff with a special qualification for work with patients after kidney transplantation. If anomalous values occured, the medical staff contacted the patient by phone or video conference to discuss the following treatment process. The control group was offered standard aftercare without telemedically supported case management. Of the 50 randomized patients, two patients in each group dropped out, resulting in 46 available valid datasets.

### One-year costs of medical service utilization

At three points (3, 6 and 12 months after transplantation), utilization of health care resources is recorded for every patient. For outpatient care visits at general practitioners (*N* = 194) and or medical specialists (*N* = 1829), standardized unit costs from Bock et al [2014] [17] were applied, representing costs from a societal perspective. For scheduled (*N* = 39) and unscheduled (*N* = 68) episodes of hospitalization, actual amounts of reimbursement were collected from the respective hospitals. Ambulatory visits at the outpatient department were priced according to the respective reimbursement (€270 per visit). All prices reflect 2015 values. Overall, six cost figures (*costs of general practitioner* out-patient care *visits, costs of medical specialist* out-patient care *visits, costs of scheduled in-hospital care, costs of unscheduled in-hospital care, costs of ambulatory visits at the outpatient department and total costs of care*) were calculated for each observation period and patient.

### Costs of telemedically supported case management

Within the statutory health insurance in Germany, telemedically supported case management is not an element of standard care. A reimbursement by sickness funds is possible by using individual case requests, only. A hypothetical number of 20 patients receiving telemedically supported case management for a one year period is assumed to require an additional 50% nurse position (€28,500) with a suitably equipped work place (€500) and internal server provision (€1,024). In addition, patient-variable costs for a Touch-Screen-PC (€600) and software licenses (€300 per year) are claimed. To these fixed (€30,024 for 20 hypothetical patients) and variable (€900 per patient) costs of the one year telemedical support, an extra 25% for infrastructure expenses was added.

### Statistical analysis

Skewed data is a major issue in statistical models of healthcare costs [18–20]. Beside the fact that the six cost figures were positively skewed, most of them were equal to zero during a considerable number of observation periods because patients did not see a physician and/or were not hospitalized in the respective time period. In order to accommodate these characteristics of the data, a two-part model approach was chosen for the regression analyses [21–24]. In two part models, a binary choice model is estimated for the probability of observing a zero versus positive outcome. Then, conditional on a positive outcome, an appropriate regression model is estimated for the positive outcome [25]. For part one of the applied models a logistic regression analysis was chosen to predict whether or not patients would utilize resources related to the respective costs figure. As recommended in the literature [20, 21, 24, 26–28], a generalized linear model (GLM) with the log link and gamma distribution was chosen for the second part. In order to first analyze the temporal development of the cost estimates between the two treatment groups, an interaction term between treatment group and observation period was used. The cluster option was used to address the fact that multiple monthly cost estimates are included in the dataset for the same patient. Then, the respective resource uses were summarized over the one-year period for each patient in order to analyze the treatment-related cost differences across the entire one-year period. Finally, a budget impact analysis [29] from the healthcare perspective is performed by combining the costs of a hypothetical number of 20 patients receiving telemedically supported case management with the actual results of summarized resource uses in the two groups. One-way sensitivity analyses are carried out to assess the impact that changes in a certain parameter will have on the analyses’ results. Marginal means from all regression analyses are shown on the raw scale (€ per observation period). All analyses were performed using Stata 14 (Stata Corp., Texas. USA).

## Results

### Baseline characteristics

Table 1 provides an overview of baseline patient characteristics. The medical results of the RCT this study is a part of, including more detailed statistics, have been published elsewhere [8].

**Table 1:** Patient baseline characteristics after kidney transplantation (timepoint 0)

Characteristics	Standard	Telemedical
Median age in years (range)	51 (19–66)	46 (18–59)
Male sex	47,8%	60,9%
ABO-incompatible living kidney graft	26.1%	30.4%
HLA-Mismatches ≤ 4	43.5%	47.8%
First graft	82.6%	82.6%
Postoperative complications	52.2%	47.8%
Median graft GFR in ml	57.99	53.99
(range)	(13.60–82.92)	(38,48–81,95)

### One-year costs of medical service utilization

Table 2 provides estimates regarding the different cost figures over the three observational periods. Between month 0 and month 3 after transplantation, the number of out-patient care visits is comparable between the two groups. Between month 3 and month 6, however, the group of patients with telemedically supported case management required fewer medical specialist consultations, which is associated with average cost savings of € 293.54 per patient (*p* = 0.036) for this period. Between month 6 and month 12 after transplantation, these cost savings increase to € 419.12 (*p* = 0.089). Over the entire one-year period, patients with telemedically supported case management substantially less frequently visited medical specialists, resulting in total cost savings of € 761.37 per patient (*p* = 0.048). As far as general practitioner visits are concerned, on the other hand, the costs are relatively identical between both groups (*p* = 0.879).

**Table 2:** Comparison of cost across the six cost figures

	Month 0‐3	Month 3‐6	Month 6‐12	Total cost	*p*‐value (total costs)
Costs of primary care physician visits:					
Standard	€13.39	€20.44	€35.94	€69.77	
Telemedical	€11.28	€21.85	€33.83	€66.95	
Difference	€2.11	-€1.41	€2.11	€2.82	0.879
Costs of specialist consultant visits:					
Standard	€650.31	€746.58	€1,053.31	€2,450.20	
Telemedical	€601.60	€453.04	€634.20	€1,688.83	
Difference	€48.72	€293.54	€419.12	€761.37	0.048
Costs of unscheduled in‐hospital care:					
Standard	€2,376.24	€1,813.49	€1,245.33	€5,435.06	
Telemedical	€1,463.60	€190.78	€363.23	€2,017.60	
Difference	€912.64	€1,622.72	€882.10	€3,417.46	0.003
Costs of scheduled in‐hospital care:					
Standard	€885.77	€338.14	€640.82	€1,920.36	
Telemedical	€843.79	€39.66	€72.60	€956.05	
Difference	€41.98	€298.47	€568.22	€964.31	0.073
Costs of amulatory visists at the university outpatient department:					
Standard	€246.52	€199.57	€211.30	€657.39	
Telemedical	€258.26	€246.52	€270.00	€774.78	
Difference	-€11.74	-€46.96	-€58.70	-€117.39	
Total costs of care:					
Standard	€4,172.23	€3,118.21	€3,158.84	€10,449.28	
Telemedical	€3,178.52	€951.84	€1,373.85	€5,504.21	
Difference	€993.71	€2,166.36	€1,784.99	€4,945.07	

**Fig. 1:**
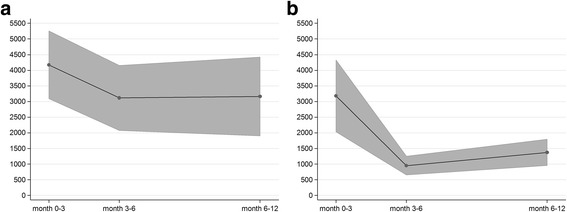
Total costs of care. **a** standard aftercare and **b** telemedically supported case management

As a result of early diagnoses and short delay between the first onset of symptoms and initiation of treatment, telemedically supported case management is associated with substantial cost savings related to unscheduled hospitalizations. The associated cost savings are highest between month 3 and month 6 after transplantation and add up to savings of € 3417.46 (*p* = 0.003) for the entire one-year period. The costs of scheduled in-hospital care, in contrast, are less affected by telemedically supported case management (cost savings of € 964.31 over the entire one-year period, *p* = 0.073). On the other hand, patients with telemedically supported case management more often applied for ambulatory visits at the university outpatient department, resulting in additional costs of € 117.39 (*p* = 0.035) for the entire one-year period. Overall, telemedically supported case management is clearly associated with cost savings due to less frequent and/or less intense medical service utilization during the one-year period (see Fig. 1). These savings are highest between month 3 and month 6 after transplantation (€ 2166.36, *p* < 0.001) and add up to total savings of € 4945.07 (*p* < 0.001) for the entire one-year period. Overall, patients in the standard aftercare group are associated, on average, with €10,449.28 of on-year medical service utilization costs while patients with telemedically supported case management are associated with €5,504.21 of on-year medical service utilization costs.

### Budget impact analysis^1^ of telemedically supported case management

The main objective of our study is to calculate the budget impact of the telemedically supported case management from the healthcare perspective. As a first step our analysis combines the costs of a hypothetical number of 20 patients receiving telemedically supported case management with the actual results of summarized resource uses in the two groups. As a result, costs of telemedically supported case management are estimated at €3,001.5 for a single patient. Although being hypothetical, this cost figure equals the amount of reimbursement from the different sickness funds using individual case requests. After combining this reimbursement (€3,001.5) with the costs of medical service utilization (€5,504.21, see above), the one-year costs in the telemedically supported case management group add up to €8,505.71. In comparison to the costs of medical service utilization in the standard aftercare group (€10,449.28, see above), telemedically supported case management may still be associated with lower costs (mean difference: €1,943.57), but this result is not statistically significant (*p* = 0.128).

**Fig. 2:**
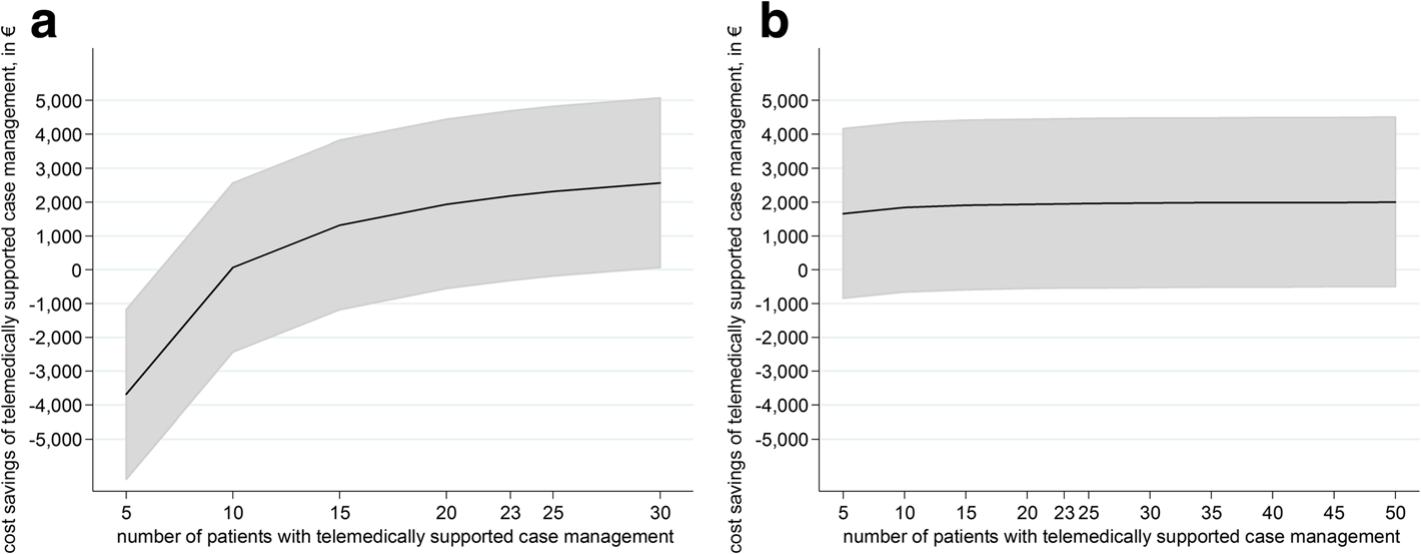
Cost savings per patient. **a** …keeping the fixed costs at a constant level and **b** …keeping the share of personnel expenses flexible

As a first sensitivity analysis, we calculate the net savings of our telemedically supported case management program depending on the number of patients participating. Due to the effect of fixed cost degression the program becomes cost-neutral at around ten participating patients, and was beneficial at 15 patients (see Fig. 2). After an initial rapid increase, the increase of savings begins to level off, increasing from €67 at a program size of ten patients to €1318 at 15 patients, but only from €2318 to €2569 from 25 to 30 patients. The results are projected to become statistically significant (*p* < 0.05) at a program size of 30 patients. Please note that the results for more than 20 patients imply the assumption that an additional 50% nurse position may can provide telemedically supported case management to more than 20 patients.

As a second sensitivity analysis, we kept the share of personnel expenses (an additional 50% nurse position for 20 patients) flexible. We assumed a 2.5% nurse position necessary for every additional patient with telemedically supported case management (which is equal to €1,781.25). As shown in Fig. 2, the program now becomes beneficial even from a very low number of patients. Please note that these results imply the assumption that the nurse position may be easily increased and decreased relative to the number of patients with telemedically supported case management. All results of the sensitivity analyses are shown in Table 3.

**Table 3:** Budget impact analysis

	Hypothetical n	Standard	Telemedical	Difference	*p*-value
Sensitivity analysis 1: 5‐30 patients, fixed costs at a constant level (in € and per patient)					
Base case	5 patients	€ 10449.28	€ 14135.21	‐€3685.93	0.004
	10 patients	€ 10449.28	€ 10382.21	€ 67.07	0.958
	15 patients	€ 10449.28	€ 9131.21	€ 1318.07	0.301
	20 patients	€ 10449.28	€ 8505.71	€ 1943.57	0.128
	23 patients	€ 10449.28	€ 8260.95	€ 2188.33	0.086
	25 patients	€ 10449.28	€ 8130.41	€ 2318.87	0.069
	30 patients	€ 10449.28	€ 7880.21	€ 2569.07	0.044
Sensitivity analysis 2: 5‐50 patients, keeping personal cost flexible (in € and per patient)					
Base case	5 patients	€ 10449.28	€ 8791.46	€ 1657.82	0.194
	10 patients	€ 10449.28	€ 8600.96	€ 1848.32	0.147
	15 patients	€ 10449.28	€ 8537.46	€ 1911.82	0.134
	20 patients	€ 10449.28	€ 8505.71	€ 1943.57	0.128
	23 patients	€ 10449.28	€ 8493.28	€ 1955.99	0.125
	25 patients	€ 10449.28	€ 8486.66	€ 1962.62	0.124
	30 patients	€ 10449.28	€ 8473.96	€ 1975.32	0.121
	35 patients	€ 10449.28	€ 8464.89	€ 1984.39	0.12
	40 patients	€ 10449.28	€ 8458.08	€ 1991.20	0.118
	45 patients	€ 10449.28	€ 8452.79	€ 1996.49	0.117
	50 patients	€ 10449.28	€ 8448.56	€ 2000.72	0.117

Finally we transfer our results to the German healthcare system. We recommend that the program should be implemented in bigger transplant centers like Freiburg, with more than 30 living donor kidney transplantations a year. The program is highly feasible and, if implemented in this way, would result in significant savings of upwards of €3,000 per patient and year of telemedically supported case management. According to EuroTransplant, a total of 645 living kidney transplantations were conducted in 38 German transplant centers in 2015 [30]. Of these, 12 centers conducted more than 20 transplantations (n ~ 407 in total). According to our results, routine implementation of telemedically supported case management in these 12 centers would result in annual savings of €791,033 according to the procedure numbers of 2015 and the results of our base case scenario.

## Discussion

Overall, the results of our study show that telemedically supported case management is associated with a substantially lower frequency of medical service utilization. As a result, one-year costs of care for these patients (€5,504.21) are roughly half the costs in the standard aftercare group (€10,449.28). Analysis of the different cost categories shows that the major drivers of these differences are episodes of unscheduled in-hospital care. This confirms the main rationale behind the study: An early diagnosis and short delay between the first onset of symptoms and initiation of treatment correlates with substantially lower medical service utilization. Our telemedicine intervention has shown to be effective at improving several important outcome measures of living donor kidney transplantation. The intervention has shown promise for better medical and disease-specific outcomes as well as saving costs – a win-win situation.

From the healthcare perspective, an implementation of the program in the 12 biggest transplant centers in Germany would result in annual net savings of €791,033 per year. However, the current reimbursement practice requires requests to be made for each case separately to each of the 117 German statutory health insurances. This may be seen as the main hurdle for implementing telemedically supported case management in other centers for the following reasons: Firstly, the high number of requests goes hand in hand with high negotiation and administrative costs. Secondly, from the point of view of the supplier of telemedicine aftercare infrastructure (in this case the transplantation center) the reimbursement and therefore the utilization of the investment is a priori uncertain. Thus, we propose to reimburse telemedically supported case management by sickness funds in a routine manner. Political action is needed in order to unlock these potential patient- and societal-level benefits.

Our study avoids several of the problems that are otherwise common in studies of the cost-efficacy of telemedicine [9, 10]. However, please note that the results of the present study are still subject to a number of limitations. First of all, 23 patients in each group is a very limited number of patients given the wide variation in cost measures. As a result, measures of significance in many cases do not reach the usual level of significance (*p* < 0.05) and results should be interpreted with the according caution. Also, the patient population was not large enough to properly control for socioeconomic background. For example, elderly patients might still be less comfortable with using telemedical devices, which could result in this or other patient populations receiving better results from traditional aftercare programs. On the other hand this means that taking such factors into account in the patient selection process, or making adjustments to our telemedical equipment and techniques to improve accessibility for any patients currently struggling to make best use of the equipment, might further improve the already significant impact of telemedical support on patient outcomes and costs we measured.

Secondly, collection of patient-level cost data during follow-up is a resource intensive exercise. In order to simplify this process, we decided to apply standardized unit cost for visits at primary care physicians, specialist or ambulatory visits at the university outpatient department [17]. Presumably, this simplification might underestimate the true ambulatory expenditures as ambulatory care of transplant patients is exceptionally resource intensive. In addition, this simplification completely ignores medical treatment expenditures. Immunosuppressive regimes in renal transplant patients are costly, but adherence with medication may be considered of major importance for post-procedural outcomes [31]. Probably, adherence with medication is also improved by telemedically supported case management due to the fact that unnecessary changes in the drug therapy can be avoided. Recent regulatory changes towards an exclusion of immunosuppressive regimes from aut idem regulations in Germany underline the necessity of medication adherence among transplant patients [32, 33].

## Conclusions

Our random controlled trial, covering about 7% of all living kidney transplantations conducted in Germany over the trial period, shows that the introduction of telemedically supported case management results not only in significant improvements in medical and disease-specific outcomes, but also a considerable potential for cost savings within the first year after transplantation, mainly by avoiding episodes of unscheduled in-hospital care.

Due to the effect of fixed cost degression we recommend that the program should be implemented in medium and high volume transplant centers. This would result in annual savings of almost €800,000 for the German healthcare system for living kidney transplantations alone.

From a broader perspective the major hurdle still standing in the way of widespread introduction of the telemedicine aftercare technique at the interface between the outpatient and inpatient sectors is the current reimbursement practice, in which requests have to be made for each case separately to each of the German statutory health insurances, representing a significant administrative hurdle. Hence, provision would have to be made for telemedically supported case management to be reimbursable by sickness funds in a routine manner.

## Abbreviation

GLM: Generalized linear model

## Footnotes

1 Additional to our cost perspective the results also can be interpreted as net benefit to the patients, since their quality of life and health is improved by the earlier diagnosis and short delay between the first onset of symptoms and initiation of treatment resulting from the telemedical supported case management program.
